# Analysis of Coinfections with A/H1N1 Strain Variants among Pigs in Poland by Multitemperature Single-Strand Conformational Polymorphism

**DOI:** 10.1155/2015/535908

**Published:** 2015-04-15

**Authors:** Krzysztof Lepek, Beata Pajak, Lukasz Rabalski, Kinga Urbaniak, Krzysztof Kucharczyk, Iwona Markowska-Daniel, Boguslaw Szewczyk

**Affiliations:** ^1^Laboratory of Recombinant Vaccines, Intercollegiate Faculty of Biotechnology, University of Gdansk and Medical University of Gdansk, Kladki 24, 80-822 Gdansk, Poland; ^2^BioVectis Ltd., Pawinskiego 5A/D, 02-106 Warsaw, Poland; ^3^Electron Microscopy Platform, Mossakowski Medical Research Centre, Pawinskiego 5, 02-106 Warsaw, Poland; ^4^Department of Physiological Sciences, Faculty of Veterinary Medicine, Warsaw University of Life Sciences (SGGW), Nowoursynowska 159, 02-776 Warsaw, Poland; ^5^Department of Swine Diseases, The National Veterinary Research Institute, Partyzantów 57, 24-100 Pulawy, Poland

## Abstract

Monitoring and control of infections are key parts of surveillance systems and epidemiological risk prevention. In the case of influenza A viruses (IAVs), which show high variability, a wide range of hosts, and a potential of reassortment between different strains, it is essential to study not only people, but also animals living in the immediate surroundings. If understated, the animals might become a source of newly formed infectious strains with a pandemic potential. Special attention should be focused on pigs, because of the receptors specific for virus strains originating from different species, localized in their respiratory tract. Pigs are prone to mixed infections and may constitute a reservoir of potentially dangerous IAV strains resulting from genetic reassortment. It has been reported that a quadruple reassortant, A(H1N1)pdm09, can be easily transmitted from humans to pigs and serve as a donor of genetic segments for new strains capable of infecting humans. Therefore, it is highly desirable to develop a simple, cost-effective, and rapid method for evaluation of IAV genetic variability. We describe a method based on multitemperature single-strand conformational polymorphism (MSSCP), using a fragment of the hemagglutinin (HA) gene, for detection of coinfections and differentiation of genetic variants of the virus, difficult to identify by conventional diagnostic.

## 1. Introduction

The influenza A virus (IAV) belongs to the Orthomyxoviridae family and is the main cause of the annual incidence of human and animal flu [1]. Due to the nature of the virus genetic material and the related phenomena of antigenic “drift” and “shift,” the disease may take the form of a seasonal wave of cases covering a large area of a particular country, a local epidemic, or a global pandemic [2].

The influenza A virus is classified based on two major glycoproteins: hemagglutinin (HA) and neuraminidase (NA) [3]. Sixteen subtypes of HA and nine subtypes of NA can be found in wild aquatic birds around the world, which are the natural reservoir of the virus. Other species, such as humans, horses, pigs, and marine mammals, can be infected with a virus from the primary reservoir, but such cases are rare. Zoonotic infections usually do not lead to an epidemic with maintained “human to human” transmission of the virus. However, such a possibility exists and may have very serious and extensive consequences for the human population [4].

In order to adapt to a new host and replicate efficiently, the virus needs to overcome species barriers and adjust to factors specific to the new host. IAV is able to do it due to the organization and processing of its genetic material [5]. The genome of IAV is subdivided into eight RNA segments encoding several viral proteins [1]. The lack of proofreading properties of the RNA polymerase gives rise to minor changes in the structure of viral proteins (in particular HA and NA). It allows the virus to escape the immune response and cause a local influenza epidemic. This phenomenon is called “antigenic drift” [6]. Another more dangerous and less predictable phenomenon responsible for the virus variability is called “antigenic shift.” It is a sudden and significant change of major virus antigens, caused by reassortment of genome segments during coinfection of one host with more than one virus strain. It can lead to the emergence of a dangerous, potentially pandemic strain, capable of efficient infection and transmission between humans [7].

HA is one of the factors responsible for the host specificity of IAV. It recognizes receptors on the surface of epithelial cells, responsible for binding virus particles. All hemagglutinins of IAV are specific for receptors of different hosts. “Avian” strains usually recognize sialic acids linked to galactose by alpha-2,3 bonds, whereas “human” strains recognize sialic acids linked to galactose by alpha-2,6 bonds [8]. Sialic acids linked both by alpha-2,3 and alpha-2,6 bonds were found in the airways of pigs, which are therefore susceptible to infection with both “avian” and “human” strains and can serve as a mixing vessel for reassortment [9, 10]. The A(H1N1)pdm09 strain responsible for the outbreak of a pandemic in 2009 resulted from reassortment between four different IAV strains. It contained a combination of genes from human, swine, and avian influenza viruses [11]. Due to the efficient spread of the A(H1N1)pdm09 strain between humans, it has almost entirely supplanted the strain of human influenza A/H1N1 virus which used to be primarily responsible for seasonal morbidity [12]. The A(H1N1)pdm09 strain has also been detected in animals. It retained the possibility of infecting pigs and spreads quickly among the pig population in many European countries. There were many confirmed cases of human-originated animal infections [13–21]. The cases of swine-originating H3N2v infections among humans in the USA prove that the emergence of a new virus strain capable of efficient transmission between humans is a serious threat [22].

Current diagnostics and IAV genotyping in pigs are based on widely used serological methods, such as the hemagglutinin inhibition assay (HI), neuraminidase inhibition assay (NI), and a broad range of molecular methods based on reverse transcriptase polymerase chain reaction (RT-PCR), multiplex RT-PCR (MRT-PCR), and real time RT-PCR (RRT-PCR) [23–28]. Unfortunately, the inhibition assays are not specific enough for detection of different genetic variants of IAV belonging to the same subtype. On the other hand, PCR based methods are vulnerable to minor changes in nucleotide sequences, especially when using specific hybridization probes, or melting curve analysis [23, 29–31].

An alternative method for rapid identification of minor genetic variants that might escape detection by popular diagnostic methods is multitemperature single-strand conformational polymorphism (MSSCP). MSSCP is based on electrophoretic separation of single-stranded DNA in native conditions, at sequentially changed gel temperature. Under such conditions, the PCR products are more likely to adopt different conformations during separation due to the differences in nucleotide sequence [32, 33].

Because the IAV genome undergoes constant changes, there is a demand for fast, reliable, and cost-effective methods for detection of mixed infections with quasispecies or novel genetic variants of the virus. This would help to assess the risk associated with close contacts between humans and pigs. We describe a quick and sensitive MSSCP method for monitoring the diversity of the IAV population among pigs.

## 2. Materials and Methods

### 2.1. Sample Collection

The biological material (five lung tissue samples) was collected in the Department of Swine Diseases from pigs showing influenza-like symptoms, coming from three Polish farms located in three different provinces (Lubelskie, Lodzkie, and Wielkopolskie) in years 2011–2013 (Table 1). The lung tissue was suspended in PBS buffer supplemented with penicillin (1000 U/mL), streptomycin (1 mg/mL), gentamicin (5 *μ*g/mL), and FBS (5%). The tissue samples were then homogenized and used for RNA isolation. All samples were stored at −80°C for further investigation.

### 2.2. Total RNA Extraction

The viral RNA was extracted from the tissue homogenate with the use of QIAamp Viral RNA Mini Kit (QIAGEN, Valencia, CA), according to the manufacturer's instruction.

### 2.3. Virus Detection and Genotyping

The RNA was used for partial amplification of the matrix gene of the swine influenza virus through RRT-PCR using QuantiTect Probe RT-PCR Kit (QIAGEN, catalogue number 204443, Valencia, CA). The primer sequences, the probe, and the PCR protocol were described elsewhere [34]. In order to determine the virus subtype, the RNA samples considered as positive, based on RRT-PCR, were simultaneously examined in two conventional MRT-PCRs, in order to identify the* HA* and* NA* genes. The first MRT-PCR distinguished between the* HA1* of human and avian origin and* HA3* genes of swine origin. The second MRT-PCR, with 2 sets of primers, was carried out to detect fragments of the* NA1* and* NA2* genes. The MRT-PCRs were performed using One Step RT-PCR Kit (QIAGEN, catalogue number 210212, Valencia, CA), according to Chiapponi et al. [35]. The RRT-PCR and MRT-PCRs were conducted using Stratagene MX3005P (Agilent Technologies, USA) and T3 Thermocycler (Biometra, Germany), respectively.

### 2.4. cDNA Synthesis

The extracted viral RNA was used for cDNA synthesis with the ThermoScript RT-PCR System for First-Strand cDNA Synthesis (Invitrogen, catalogue number 11146-024). Instead of using the supplied random primers for amplification of the whole genome of IAV, we used the Uni12 universal primer described elsewhere [36]. A mixture of the viral RNA template, Uni12 universal primer, and 10 mM dNTP Mix (12 *μ*L in total) was incubated at 65°C for 5 min and then chilled on ice. A cDNA synthesis buffer, 0.1 M DTT, RNaseOUT, ThermoScript reverse transcriptase, and water, was then added, and the mixture was incubated at 65°C for 60 min, followed by termination of the reaction at 85°C for 5 min. The cDNA was later used in PCR assays.

### 2.5. Amplification of a Hemagglutinin Gene Fragment by PCR

The cDNA was diluted (10x) before use for amplification of an* HA* gene fragment. The sequences of the specific primers were H1msscp1 (5′-AGTAACACACTCTGT-3′) and H1msscp2 (5′-ACAATGTAGGACCATGA-3′). The primers were synthesized by GENOMED S.A. (Warsaw, Poland). The reaction was performed using KAPA HiFi HotStart ReadyMix PCR Kit (Kappa Biosystems, catalogue number KK2601). Water, primers (10 mM), and 1 *μ*L of cDNA solution were added to the HotStart ReadyMix, in a total volume of 25 *μ*L. The assay was performed in T-personal 48 Thermocycler (Biometra, Germany) as follows: initial denaturation at 98°C for 5 min, followed by 40 cycles of denaturation at 98°C for 20 s, annealing at 56°C for 15 s, and extension at 72°C for 15 s. The reaction ended with a final extension step at 72°C for 5 min. For Sanger sequencing, the PCR products were subjected to electrophoretic analysis in 1% agarose gel, in TAE buffer (20 mM sodium acetate, 1 mM EDTA, and 40 mM TRIS, pH adjusted to 7.2), using SimplySafe (Eurx, Poland), and then purified on silica gel columns (Gel-Out Concentrator, A&A Biotechnology, Poland).

### 2.6. MSSCP Analysis

The PCR products were screened by MSSCP [33] for the genetic diversity of* HA* amplicons. The PCR products were heat-denatured and shortly chilled, and ssDNA conformers were resolved in native conditions. The analysis was performed using the DNA*Pointer* System in 0.5x TBE buffer. The MSSCP conditions were optimized and the electrophoresis was performed on a polyacrylamide gel (10% T, 3.3% C), in 0.75x TBE buffer, at 40 W. The temperature profile of the electrophoresis was 15–10–5°C. Before applying samples onto the gel, 10 min of preelectrophoresis (40 W at 35°C) was performed. The samples were maintained for 10 min at 100 V for concentration and then separated by MSSCP. The separated ssDNA bands were visualized by silver nitrate staining (Silver Stain DNA Kit, BioVectis, catalogue number 200-101). The ssDNA bands of altered MSSCP mobility, compared to the reference sample, were cut out. The ssDNA was eluted, reamplified (using the primers and PCR conditions described above), purified with exonuclease I and shrimp alkaline phosphatase (Fermentas, catalogue numbers EN0581 and EF0511), and analyzed by Sanger sequencing (3730xl DNA Analyzer, Applied Biosystems, Carlsbad, CA, USA).

### 2.7. Clonal Selection of Mixed Genetic Variants

The amplified* HA* gene fragments from the five isolates (sw1–5) were subjected to clonal selection of mixed genetic variants. The PCR products were resolved on 1% agarose gel in TAE buffer (20 mM sodium acetate, 1 mM EDTA, and 40 mM TRIS, pH adjusted to 7.2) with SimplySafe (Eurx, Poland) and purified on silica gel columns (Gel-Out Concentrator, A&A Biotechnology, Poland). Next, they were cloned into the pJet1.2 plasmid vector (Clone JET PCR Cloning Kit, Thermo Scientific, USA), according to the manufacturer's procedure. Positive recombinant plasmids were used for transformation of TOP10* E. coli* competent cells (Life Technologies, USA). Selected colonies were isolated and were grown in a liquid medium. The recombinant plasmids were isolated from the liquid medium on silica gel columns (Plasmid Mini Concentrator, A&A Biotechnology, Poland) and used as a template for PCR amplification of an* HA* gene fragment prior to MSSCP.

### 2.8. Bioinformatical and Phylogenetic Analysis

The results of Sanger sequencing were used for a BLAST search in the GenBank nucleotide database. The best matching hits (with their identity percentage) are presented in Table 2. The nucleotide sequences were aligned in Geneious 7 [37] software by using the MAFT tool [38] with default settings. The obtained results were then exported to MEGA 6 software [39] for phylogenetic analysis, where the evolutionary history was inferred using the Minimum Evolution method [40]. The confidence probability was computed using a bootstrap test with 1000 replicates. The phylogenetic trees were drawn to scale, using the same units for branch lengths and the evolutionary distances used to infer the phylogenetic trees. The evolutionary distances were computed using the Maximum Composite Likelihood method [41] and the results are expressed in base substitutions per site. The ME tree was searched for using the close-neighbor-interchange (CNI) algorithm [42] at the search level of 1. To generate the initial tree, the neighbor-joining algorithm [43] was used.

## 3. Results

In years 2011–2013, the National Veterinary Research Institute collected five environmental samples (lung tissue) on farms in Poland, from pigs with influenza-like symptoms. The presence of swine IAV was confirmed with RRT-PCR, and the genotype of the virus was determined by MRT-PCR. All isolates were characterized as swine “avian-like” H1N1 (Table 1).

**Figure 1:**
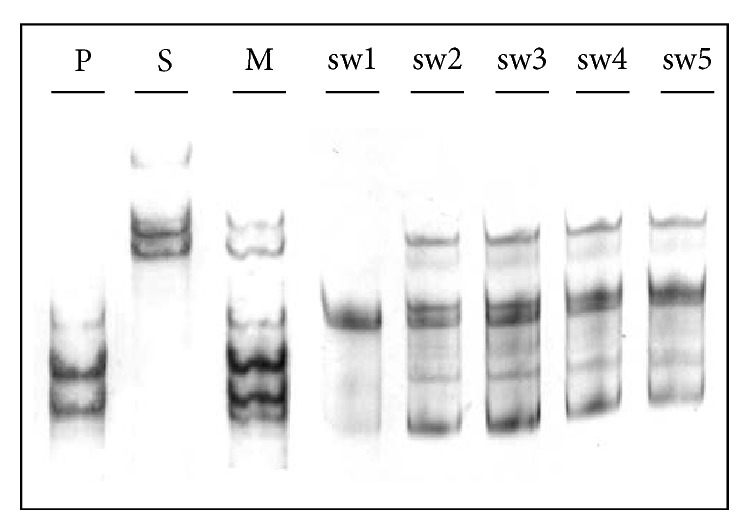
MSSCP-based identification of mixed infections in five swine samples collected from three farms in Poland in 2011–2013. The following samples were amplified by PCR and their corresponding ssDNA was separated on a 10% polyacrylamide gel using MSSCP method under optimum electrophoretic conditions: a hemagglutinin gene fragment from the five isolates (sw1–sw5), a reference pandemic A/Mexico/4486/09 strain (P) and a seasonal A/Brisbane/59/2007 strain (S), and a sample from a patient diagnosed with a coinfection with both the pandemic and seasonal strains (M). The DNA was visualized by silver staining. The electrophoretic profile of the analyzed swine samples sw2–sw5 is similar to the coinfection profile.

To check whether the IAVs from the collected isolates were of human origin and/or to verify the possibility of mixed infections, the RNA isolated from the samples was sent for further analysis. In the Laboratory of Recombinant Vaccines, the RNA was analyzed using an MSSCP-based minor variant enrichment procedure, which utilizes an* HA* gene fragment encompassing nucleotides 125 to 302. This fragment corresponds to the HA1 polypeptide region starting 25 amino acids after the N-terminal signal peptide of HA. The MSSCP analysis allows easy detection and distinction of infections and coinfections with the pandemic A(H1N1)pdm09 and seasonal A(H1N1) strains in humans. The five isolates from pigs were used to synthesize the first strand of cDNA by RT-PCR. Next, amplification using specific primers was performed on the fragments of* HA* gene from all isolates; two reference strains, pandemic (A/Mexico/4486/09) and seasonal (A/Brisbane/59/2007); and one sample from a patient previously diagnosed with a coinfection. The PCR products were denatured and native electrophoresis was performed under optimal MSSCP electrophoresis conditions (15°C–10°C–5°C, 450 Vxh/per phase, 10% polyacrylamide gel). The separated ssDNA was then visualized with a silver stain. The results of MSSCP separation are shown in Figure 1.

**Figure 2:**
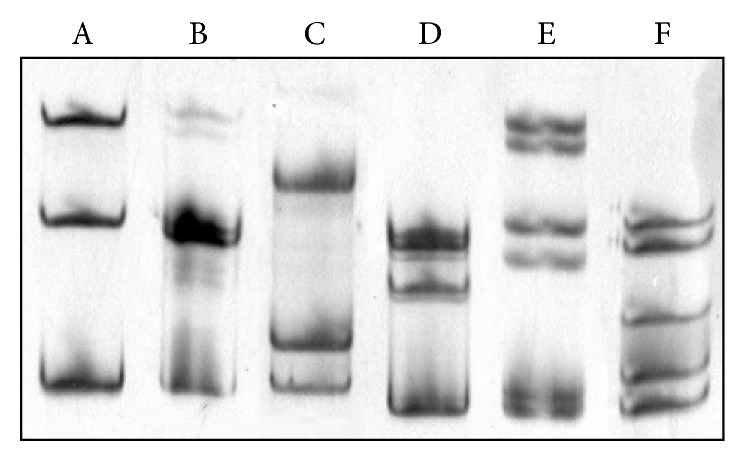
Distinct genetic variants (A–F) of the influenza A/H1N1 virus revealed by MSSCP of the collected isolates after clonal selection. DNA fragments corresponding to unique band patterns that occurred repeatedly during analyses of clones from five swine isolates were denatured and were separated on 10% polyacrylamide gel using MSSCP method under optimal electrophoretic conditions. DNA was visualized by silver staining.

It is evident that the electrophoretic profiles of the swine samples (sw1–sw5) do not match the profiles of seasonal (S) or pandemic (P) reference strains. Furthermore, the swine isolates sw2–sw5 are very similar to each other and have a similar number of ssDNA bands, as the coinfection sample (M). This result might suggest multistrain and/or quasispecies infections. To verify this hypothesis, the amplified* HA* gene fragments from the swine isolates were sequenced by using the Sanger method (data not shown). The direct sequencing confirmed that the genetic material was not uniform within the isolates. Differences in the nucleotide sequence might indicate the presence of at least two genetic variants isolated from a single individual. To address this issue, we performed clonal selection in bacterial cells. After clonal selection, we chose twenty colonies from each isolate for screening. We purified recombinant plasmids from the isolates and used them as templates for the amplification of a specific* HA* gene fragment. After MSSCP is performed on all the clones (data not shown), we distinguished six unique band patterns that occurred repeatedly during separation. To show them in one analysis, selected DNA fragments corresponding to unique band patterns were denatured, subjected to MSSCP analysis and visualized by silver staining. Six most common genetic variants with distinct electrophoretic band patterns marked with capital letters from A to F are presented in Figure 2.

To evaluate the differences in the nucleotide sequence between the individual genetic variants, all clones showing distinct band patterns were sequenced by the Sanger method. The results of a BLAST analysis of the clonally selected A/H1N1 isolates are shown in Table 2.

Based on the phylogenetic relationship, the clones were assigned to three groups (I, II, and III, with letters a, b, c, and d marking individual clones within a group). To determine the phylogenetic relationship, we compared 121nt long fragments of the selected clones with seven distant strains of IAV (shown in bold in Table 3).

**Figure 3:**
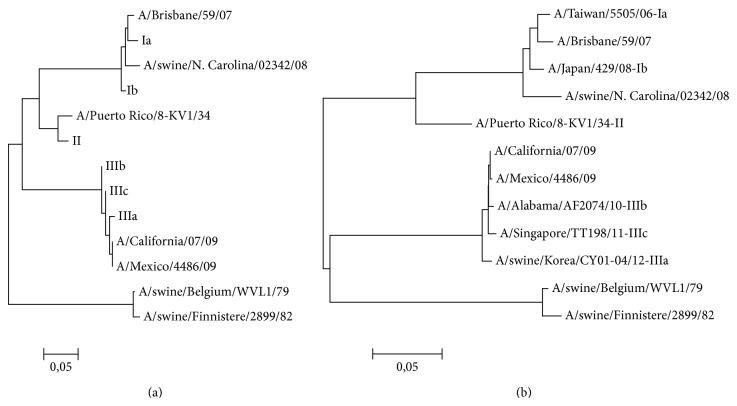
Evolutionary relationships of IAV strains. (a) shows 123nt fragments from the clonally selected isolates and the strains marked in bold in Table 3. (b) shows 706nt fragments of all the strains from Table 3.

The generated phylogenetic tree (Figure 3(a)) shows a close relationship between group I and the strains isolated in years 2006–2008, which included a classical swine A/H1N1 virus, prevalent in North America, and a human A/H1N1 lineage, predominant at the time. On the other hand, group III showed the closest relationship with strains isolated after 2009, corresponding to the pandemic A/H1N1 lineage, which has been reported in pigs since its introduction in 2009. The results for group II were surprising, because the genetic variant assigned to this group was closely related to the A/Puerto Rico/8-KV/34 strain. All the analyzed genetic variants were distant from the A/Swine/Belgium/WVL/79 and A/Swine/Finnistere/2899/82 strains representing the European swine A/H1N1 lineage of avian origin. To support our findings, we inferred the evolutionary history based on a 706nt long alignment of the 12 reference IAV strains presented in Table 3. The tree in Figure 3(b) presents a similar arrangement of branches and distances, which confirms the tree based on a shorter alignment of the selected clones (Figure 3(a)).

## 4. Discussion

In April 2009, in Mexico, the first cases of human infections with a new influenza A/H1N1 virus were reported. Shortly afterwards, the new strain has spread very quickly among humans all over the world, causing millions of infections. As a result, in June 2009, the World Health Organization (WHO) declared a state of global pandemic due to emergence of a novel strain of IAV. In most populations, the disease caused mild or moderate symptoms and the mortality rate was relatively low [44–46]. The A(H1N1)pdm09 strain responsible for this outbreak was a result of a genetic rearrangement between a human A/H3N2, avian A/H1N1, and classical swine A/H1N1 triple reassortant and an Eurasian “avian-like” swine A/H1N1 virus [11, 46, 47]. Such reassortment of influenza virus strains illustrated their potential for crossing transspecies barriers [12, 15, 48]. Until June 2010, twenty-three countries reported confirmed cases of A(H1N1)pdm09 infection in animals, mostly in pigs [12]. The comparison of the sequences of the viral isolates from animals, obtained during the epidemic, and the human variant of the A(H1N1)pdm09 strain detected at the same locations clearly showed a strong homology in all cases. It proved the circulation of the virus among the populations of humans and pigs [15, 16]. A monitoring research system used in Norway for a pig herd free from the “swine flu” virus has shown several infections with A(H1N1)pdm09 since October 2009. Many of the infected pigs had previous contact with people diagnosed with A(H1N1)pdm09 or influenza-like illness [17].

The reassortment potential of the A(H1N1)pdm09 virus has been proven by zoonotic transmissions of swine strains to humans. In 2011 and 2012, more than three hundred cases of influenza in humans were reported in the USA, which were caused by the A/H3N2v swine-originating virus that acquired the *M* gene from the A(H1N1)pdm09 strain. Most infections with the A/H3N2v strain were associated with a man-to-pig exposure, but there were also cases of transmission between two and sometimes three persons. It was probably due to the acquisition of the M gene, which is also thought to be responsible for the improved transmission and replication of the virus in animal models and cell cultures [49–53].

The WHO, the Food and Agriculture Organization of United Nations (FAO), and the World Organization for Animal Health (OIE) issued many reports about mixed infections between humans and animals, stressing the gravity of the problem. All the organizations recommended some procedures in order to minimize the mixing of different strains of the virus. They also clearly stated that the adaptation of “human” viruses among swine populations will create a potential for reassortment with other viruses of “swine” or “avian” origin and emergence of a novel, more virulent strain [12, 54, 55]. Therefore, it is necessary to put more effort in monitoring, diagnostics, and control of influenza virus infections in animals (especially pigs), to acquire data and evaluate the risk of the emergence of a dangerous human and/or animal virus strain.

There are many molecular techniques for detection and subtyping of IAVs. Most of them use PCR for amplification of specific nucleic acid sequences [56]. These techniques include, for example, RT-PCR, real time PCR, and RRT-PCR. They are able to detect and differentiate specific strains, lineages, and subtypes of IAV, mainly through amplification of the* HA*,* NA*, and *M* gene fragments, often in a multiplex approach [24–28]. Despite all of the advantages of these methods, some diagnostic issues remain unresolved. Real time PCR often uses probe or primer hybridization, or endonuclease cleavage, frequently producing false negative results, because of mismatches in the specific attachment region [29, 30]. Moreover, the analysis of standard or high resolution melting curves fails to detect quasispecies and/or multistrain infections, unless a long and expensive preanalysis is performed [31]. Real time PCR followed by melting temperature analysis allowed Dhiman et al. [30] to differentiate three subtypes of the influenza virus: A/H1N1v, A/H1N1, and A/H3N2. However, there were 19 strains of A/H1N1v with a melting temperature outside the range validated for that subtype [30]. On the other hand, next generation sequencing (NGS) is rather rarely used for the influenza virus diagnostics, but it offers a large amount of data and an insight into the whole genome sequence. According to Ghedin et al. [57], NGS detected a mixed infection with three different genetic variants from two distinct classes of the A(H1N1)pdm09 virus. It should be noted that the NGS method is expensive and highly demanding and needs extensive bioinformatic analysis. Therefore, it is not really suitable for epidemiological research at the moment.

One of the methods that allows detecting quasispecies and multistrain IAV infections, as well as differentiating distinct genetic variants of the virus, is MSSCP. It is an electrophoretic technique separating denatured PCR products according to their single-stranded secondary structure [32, 33]. This method has been previously used for genetic characterization of a number of virus groups [58–60]. In our previous studies, MSSCP appeared to be very effective in detecting both mixed infections with different strains of IAV in humans and mutations in the gene regions responsible for the interaction with antibodies and evading the host immune response [61, 62].

In the present study, we applied an MSSCP-based method for the analysis of isolates from pigs with influenza-like symptoms. The results clearly show that this method can be used for detection of mixed infections and is a useful tool for differentiation of specific genetic variants of the virus circulating among pigs. The described method has been validated by numerous repetitions and the presence of controls is recommended for technical verification of electrophoretic separation but not required for comparison in every analysis. MSSCP can be used for initial screening of animal infections, instead of a detailed genetic analysis, and serve as an alternative to time consuming and expensive methods like, for example, NGS and RRT-PCR. The initial clonal selection and Sanger sequencing of analyzed fragments are not required in conventional analysis, but they can serve as additional tools to investigate sequence complexity of detected variants.

The phylogenetic analysis of the genetic variants revealed by MSSCP showed that the viruses can be divided into three groups. Two of them correspond very well (a 100%-99% BLAST hits identity) to the North American classical swine lineage together with the human A/H1N1 lineage (group I) and the pandemic A/H1N1/2009 lineage (group III). Group II showed a close relation (95% BLAST hits) to the A/Puerto Rico/8-KV1/34 strain, which derives from a well-known and widely used reference strain for the human A/H1N1 lineage, which circulated during the first half of the twentieth century. Because the 121nt fragment can be considered too short to predict a homology between the isolates, we extended the analysis to 706nt fragments, based on highly similar BLAST hits (100-99% identity), which supported our previous findings. It is noteworthy that neither of the genetic variants subjected to the evolutionary relationship analysis was placed in vicinity of the avian-originating, European, swine A/H1N1 lineage, to which all of the isolates were assigned after genotyping using MRT-PCR. This observation highlights the fact that standard PCR methods are not always suitable for detection of coinfections and minor genetic variants. It also reaffirms our belief in the usefulness of MSSCP-based diagnostics.

## 5. Conclusions

The constant genetic evolution of IAV is a major risk for public and animal health. An ongoing reassortment process and small changes in sequence give rise to new IAV strains, capable of overcoming species barriers. Therefore, we need to investigate the genetic variability of the viruses circulating among humans and animals, in order to prevent or detect the emergence of highly pathogenic viral strains. Many routine diagnostic methods fail to detect quasispecies or multistrain infections, due to limited sensitivity or false negative results. We describe results proving that a native electrophoretic separation of PCR products at strictly controlled temperature (MSSCP) allows detection of coinfections and differentiation of novel genetic variants of IAV in animal samples. This method is an inexpensive, sensitive, and convenient way to determine variations of influenza strains and can be a very useful screening tool for epidemiological studies.

## Figures and Tables

**Table 1:** Sample information and genotyping results.

Isolate	Collected specimen	Geographical location of isolation (province)	RRT-PCR (Ct value)	MRT-PCR	
sw1	Lung	Lubelskie	19.38	H1 avian	N1
sw2	Lung	Lubelskie	22.66	H1 avian	N1
sw3	Lung	Wielkopolskie	26.28	H1 avian	N1
sw4	Lung	Wielkopolskie	24.00	H1 avian	N1
sw5	Lung	Lodzkie	26.40	H1 avian	N1

**Table 2:** BLAST hits for the clonally selected sequences with specific band patterns.

Clone/pattern	Phylogenetic group	BLAST hit acc. number	BLAST hit year of isolation	BLAST hit identity [%]	BLAST hit strain name
A	Ia	HQ291878	2006	100	A/Taiwan/5505/06
E	Ib	KC457584	2008	100	A/Japan/429/08
B	II	CY084118	1934	95	A/Puerto Rico/8-KV1/34
F	IIIa	KC471369	2012	99	A/swine/Korea/CY01-04/12
D	IIIb	CY069737	2010	100	A/Alabama/AF2074/10
C	IIIc	CY124801	2011	99	A/Singapore/TT198/11

**Table 3:** IAV strains used in phylogenetic analysis. The seven strains chosen as reference are in bold. Grouping based on the similarities between the selected clones. BLAST hits are underlined.

Acc. number	IAV strain name
HQ291878	A/Taiwan/5505/06-Ia
KC457584	A/Japan/429/08-Ib
**CY084118**	**A/Puerto Rico/8-KV1/34-II**
KC471369	A/swine/Korea/CY01-04/12-IIIa
CY069737	A/Alabama/AF2074/10-IIIb
CY124801	A/Singapore/TT198/11-IIIc
**CY163864.1**	**A/Brisbane/59/07**
**KF009554**	**A/California/07/09**
**GQ162202.1**	**A/Mexico/4486/09**
**CY037898.1**	**A/swine/Belgium/WVL1/79**
**CY116348**	**A/swine/Finnistere/2899/82**
**CY082832**	**A/swine/N. Carolina/02342/08**
